# Vitamin Supplements Improve a Model Form of Muscular Dystrophy

**DOI:** 10.1371/journal.pbio.1001410

**Published:** 2012-10-23

**Authors:** Richard Robinson

**Affiliations:** Freelance Science Writer, Sherborn, Massachusetts, United States of America

**Figure:**
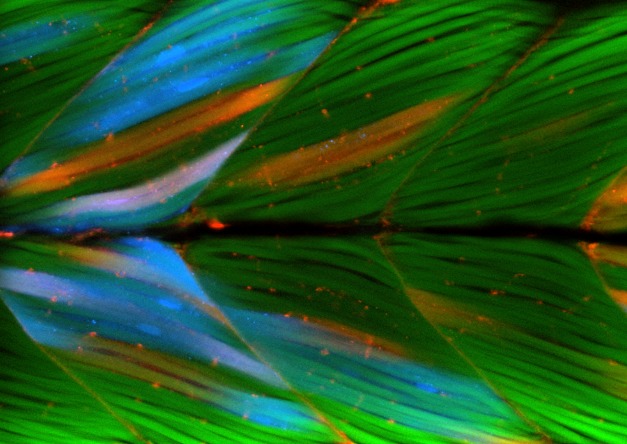
Both normal control cells (blue) and cells that lack the extracellular matrix receptor dystroglycan (red) form stable muscle cells (green), indicating that a normal extracellular matrix microenvironment plays a key role in maintaining muscle attachment.

A contracting muscle cell exerts enough force to rip itself apart in the process. That it doesn't do so is because of the strong connections it makes between its internal cytoskeleton and the external extracellular matrix (ECM). The main extracellular connector is laminin, which lies in the basement membrane (the part of the ECM closest to the cell) and links at one end to the bulk ECM and at its other end to a diverse set of receptors embedded in the muscle cell's plasma membrane.

The laminin receptors known to play crucial roles in organizing the basement membrane include the dystroglycan complex and integrin alpha7. When the receptors bind laminin, it polymerizes, forming a strong two-dimensional network across the basement membrane, organizing and strengthening it. Accordingly, inherited mutations that disrupt these receptors lead to a variety of human diseases, collectively called muscular dystrophies. In this issue of *PLOS Biology*, Michelle Goody, Clarissa Henry, and colleagues uncover the existence of a third laminin receptor complex, and show it is sensitive to vitamin supplementation.

The authors had previously shown that basement membrane formation is aided by a widespread cellular chemical, the redox coenzyme nicotine adenine dinucleotide (NAD+). In the current study, they first showed that basement membrane disorganization in dystroglycan-deficient zebrafish could be partially mitigated by providing extra NAD+, or its vitamin precursor, niacin (vitamin B3). But this rescue could not be seen in fish with mutant laminin, suggesting the beneficial effects of NAD+ might be due to its ability to promote laminin polymerization. That conclusion was strengthened by adding dystroglycan-deficient muscle cells to hosts able to make a normal basement membrane. Even in the absence of dystroglycan, most cells remained adhered to the extracellular matrix. Similarly, while absence of both dystroglycan and the second laminin receptor, integrin alpha7a, led to malformation of the myotendinous junction (which links muscle to tendon), NAD+ supplementation partially reversed this effect.

All of these results suggested the presence of a third, NAD+ sensitive laminin receptor. When the authors examined other laminin-binding proteins, they found that one, integrin alpha6 (Itga6), was upregulated in response to dystroglycan deficiency. Absence of Itga6 led to disrupted muscle development, an effect that could not be mitigated by NAD+, indicating that the two function in the same pathway.

NAD+, then, appears to promote laminin polymerization and basement membrane organization through its effects on Itga6 in the muscle cell membrane. However, the authors also found NAD+ improved that organization through another pathway as well, involving an intracellular protein called paxillin. In dystroglycan mutants, the level of paxillin at the myotendinous junction fell, but could be restored by NAD+ supplementation, partially mitigating the disorganizing effects of the loss of other receptors.

The results in this study don't merely help to clarify important aspects of muscle physiology. Loss of a well-ordered basement membrane is thought to be a common feature of the otherwise disparate muscular dystrophies, and therefore strategies to reverse that disorganization may prove to be applicable to many different diseases. The fact that a vitamin had a therapeutic effect in this simple model system provides further encouragement that the insights from this study may indicate a new route to development of effective therapies.

**Goody MF, Kelly MW, Reynolds CJ, Khalil A, Crawford BD, et al. (2012) NAD+ Biosynthesis Ameliorates a Zebrafish Model of Muscular Dystrophy. doi:10.1371/journal.pbio.1001409**

